# Structure Quality of LuFeO_3_ Epitaxial Layers Grown by Pulsed-Laser Deposition on Sapphire/Pt

**DOI:** 10.3390/ma13010061

**Published:** 2019-12-21

**Authors:** Sondes Bauer, Adriana Rodrigues, Lukáš Horák, Xiaowei Jin, Reinhard Schneider, Tilo Baumbach, Václav Holý

**Affiliations:** 1 Institute for Photon Science and Synchrotron Radiation, Karlsruhe Institute of Technology, Hermann-von-Helmholtz-Platz 1, D-76344 Eggenstein-Leopoldshafen, Germany; sondes.bauer@kit.edu (S.B.); adriana.rodriguez@kit.edu (A.R.); tilo.baumbach@kit.edu (T.B.); 2 Department of Solid State Physics, Charles University, Ke Karlovu 5, 121 16 Prague, Czech Republic; horak@karlov.mff.cuni.cz; 3 Laboratory for Electron Microscopy, Karlsruhe Institute of Technology, Engesserstr. 7, D-76131 Karlsruhe, Germany; xiaowei.jin@kit.edu (X.J.); reinhard.schneider@kit.edu (R.S.); 4 CEITEC, Masaryk University, Kotlářská 2, 611 37 Brno, Czech Republic

**Keywords:** pulsed-laser deposition, in situ X-ray diffraction, electron microscopy, multiferroics

## Abstract

Structural quality of LuFeO${}_{3}$ epitaxial layers grown by pulsed-laser deposition on sapphire substrates with and without platinum Pt interlayers has been investigated by in situ high-resolution X-ray diffraction (reciprocal-space mapping). The parameters of the structure such as size and misorientation of mosaic blocks have been determined as functions of the thickness of LuFeO${}_{3}$ during growth and for different thicknesses of platinum interlayers up to 40 nm. By means of fitting of the time-resolved X-ray reflectivity curves and by in situ X-ray diffraction measurement, we demonstrate that the LuFeO${}_{3}$ growth rate as well as the out-of-plane lattice parameter are almost independent from Pt interlayer thickness, while the in-plane LuFeO${}_{3}$ lattice parameter decreases. We reveal that, despite the different morphologies of the Pt interlayers with different thickness, LuFeO${}_{3}$ was growing as a continuous mosaic layer and the misorientation of the mosaic blocks decreases with increasing Pt thickness. The X-ray diffraction results combined with ex situ scanning electron microscopy and high-resolution transmission electron microscopy demonstrate that the Pt interlayer significantly improves the structure of LuFeO${}_{3}$ by reducing the misfit of the LuFeO${}_{3}$ lattice with respect to the material underneath.

## 1. Introduction

Hexagonal ferrites (*h*-RFeO${}_{3}$, R=Y, Dy-Lu) based oxide materials are an important category and promising candidates for information processing and storage [1,2]. The coexisting spontaneous electric and magnetic polarizations make *h*-RFeO${}_{3}$ rare-case ferroelectric ferromagnets at low temperature [3,4]. Besides the room-temperature multiferroicity and the predicted magnetoelectric effect, *h*-RFeO${}_{3}$ is a promising material for future multiferroic applications [5]. The initial driver from a technology perspective is the capability of tuning the ferromagnetism with electric fields.

Epitaxial *h*-RFeO${}_{3}$ thin films have been deposited on various substrates including Al${}_{2}$O${}_{3}$ (0001), yttrium-stabilized zirconium oxide (YSZ) (111), and Al${}_{2}$O${}_{3}$ (0001) buffered with Pt (111) layers [6,7,8,9]. All the epitaxial growth occurs along the *c*-axis of *h*-RFeO${}_{3}$ in order to satisfy the triangular symmetry matching and to maximize the effect of interface energy with the goal to stabilize the hexagonal phase in the grown thin film. The epitaxial growth of *h*-RFeO${}_{3}$ thin films and the stabilization of the hexagonal phase depend strongly on the mutual orientation of the layer and substrate lattices (epitaxial orientation) as well as on the epitaxial strain (misfit). The epitaxial orientation is determined by the minimum free energy, which is related to the bonding across the substrate-epilayer interface and to the mismatch of the substrate and layer lattices. It is recognized that, despite the triangular symmetry matching on the abovementioned substrates, there is no obvious lattice match between *h*-RFeO${}_{3}$ and Al${}_{2}$O${}_{3}$ (0001) (a=4.7602 Å), yttrium-stabilized zirconia (YSZ) (111) (a=7.30 Å), or Pt (111) (a=5.548 Å) [10].

Nevertheless, an epitaxial growth of *h*-LuFeO${}_{3}$ (LFO) could be obtained using the pulsed-laser deposition [6,7,8,9,11]. This means that the azimuthal epitaxial orientation of *h*-LuFeO${}_{3}$ films cannot be explained simply by the lattice mismatch. One should understand the interfacial structure in detail by means of structural investigations performed in situ during pulsed-laser deposition (PLD). This approach reveals how the structural setup of the layer/substrate interface would affect the structure and the morphology of the deposited *h*-LuFeO${}_{3}$.

Epitaxial strain is an extremely important issue in epitaxial thin film growth because the strain may change the properties of the epilayer, offering opportunities of material engineering; see the reviews in References [12,13], among others. For the Al${}_{2}$O${}_{3}$ (0001) substrates, the lattice mismatch of the supercell is small but the huge misfit of the lattice constant suggests weak interfacial bonding [10]. The growth of an additional Pt interlayer aims on the one side to reduce the lattice mismatch between the deposited *h*-LuFeO${}_{3}$ layer and Al${}_{2}$O${}_{3}$ (0001) substrate and on the other side to develop ferroelectric and multiferroic devices, since the Pt layer may act as the bottom electrode.

There is a considerable scientific and technological interest in the growth of epitaxial thin Pt metal film on dielectric insulators substrates like MgO (100) [14], SrTiO${}_{3}$ [15], Al${}_{2}$O${}_{3}$ (0001) [16], and ZrO${}_{2}$ [17] by various deposition methods such as PLD [14,17], magnetron sputtering [16], and molecular-beam epitaxy (MBE) [18] due to the high conductivity and oxygen stability of Pt [15]. The morphology of grown metal-on-insulator structures has been demonstrated to be strongly dependent on different growth parameters like the growth temperature, the fluence [14], deposition rate, and the thickness of the film (i.e., number of shots), especially in the case of PLD growth. An adequate combination of PLD growth parameters needs to be found to favor the layer-by-layer growth and to avoid 3D-island growth. A growth transition from two-dimensional (2D) to three-dimensional growth was found at deposition temperatures of $T_{d}>573$ K for thin Pt films deposited by PLD onto ZrO${}_{2}$ [17]. A complete coverage of the grown Pt layer was revealed for a deposition temperature smaller than 573 K [17].

X-ray diffraction analysis of *h*-LuFeO${}_{3}$ layers was restricted in former works to the acquisition of X-ray diffraction (XRD) patterns and X-ray diffraction rocking curves of a specific diffraction spot [4,5,6,7,8,9,11]. To our knowledge, there is a lack of information regarding the mosaicity in terms of misorientation and dimension of block composing the LFO crystalline layer. In situ PLD growth analysis by means of synchrotron X-ray diffraction has been demonstrated by Bauer et al. [19].

The focus of this manuscript is to study the crystalline structure (i.e., lattice parameters and mosaicity) of LFO layers in situ during PLD growth, and its dependence on the thickness of the Pt interlayer, the multiferroicity of the LFO layers (i.e., their magnetic and ferroelectric properties), will be the subject of the forthcoming paper. One of our aims is to explore the quality, morphology, interfacial strain, and chemical composition of the interface LFO/Pt using a combination of in situ analyses of two-dimensional (2D) reciprocal-space mapping (RSM) of symmetric and asymmetric reflections of LFO and an ex situ complementary characterization by scanning electron microscopy (SEM), scanning transmission electron microscopy (STEM) combined with energy-dispersive X-ray spectroscopy (EDXS), and high-resolution transmission electron microscopy (HRTEM).

## 2. Materials and Methods

### 2.1. Pulsed-Laser Deposition and In Situ X-ray Scattering

Hexagonal *h*-LuFeO${}_{3}$ film of 10 nm thickness, named Pt0, was deposited directly on an Al${}_{2}$O${}_{3}$ (0001) substrate using pulsed-laser deposition operated by a frequency quadrupled Nd:YAG laser (wavelength 266 nm) at a repetition rate of 5 Hz. Further, in four samples named Pt10, Pt20, Pt30, and P40, 10 nm thick LFO layers were deposited on Pt interlayers, the thicknesses of which varied between 10 nm and 40 nm. It should be noted here that the Pt thicknesses were determined by fitting of X-ray specular-reflection (XRR) data—see below. For the LFO deposition, totally, 6000 laser shots were always used, divided in five consecutive growth steps with the lengths of 600, 600, 600, 1200, 1200, and 1800 shots. The pulse duration of 5 ns and a single pulse energy of 25 mJ/pulse at the target were used. The values of the fluence used for the growth of the platinum interlayers and *h*-LuFeO${}_{3}$ were 2 J/cm${}^{2}$ and 1 J/cm${}^{2}$, respectively. The pulse energy was measured prior the each growth step by using a pair of optical mirrors, which deflect the laser beam either to the growth chamber or to a laser-beam detector. The PLD growth of the Pt interlayer was carried out in high vacuum with pressure $10^{-6}$ mbar and at a temperature of 300 °C while the LFO layers were deposited at a substrate temperature of 900 °C and an oxygen background pressure of 0.27 mbar. Finally, the samples were slowly cooled down from the growth temperature to room temperature with a cooling rate of 5 °C/min.

All grown films Pt0, Pt10, Pt20, Pt30, and Pt40 were investigated by XRD and XRR in situ during the PLD growth. The in situ PLD chamber was a conventional PLD chamber, which was additionally equipped with entrance and exit Beryllium windows, allowing the X-ray beam to cross the chamber and to impinge the grown film for in situ X-ray studies. The chamber was developed together with the company SURFACE systems + technology GmbH & Co. KG (Hückelhoven, Germany) (https://www.surface-tec.com/). A reflection high-energy electron diffraction system (RHEED) was used to monitor the growth, and the chamber was combined with the heavy-duty diffractometer at nano beamline on the synchrotron facility Kara; the capabilities of the in situ PLD chamber for in situ X-ray diffraction as well as for in situ diffuse scattering methods were described in detail by Bauer et al. [19].

Prior to the growth and at the growth temperature of $T_{d}=900\;{}^{\circ }\;C$, the Al${}_{2}$O${}_{3}$ (0001) substrate was aligned with respect to the X-ray beam and the 2D-RSMs of the sapphire diffraction maxima (0006) and (000.12) were recorded at room temperature (RT) and at 900 °C in order to be used as a reference for data corrections. To monitor the evolution of the crystalline structure and to determine the film thicknesses of Pt interlayer as well as the LFO layers, several XRD 2D-RSMs and XRR were acquired after the Pt growth and during the growth interruptions between the growth steps mentioned above. In the following, only LFO results will be reported; the Pt RSMs were only used for the determination of the Pt lattice parameters. An in situ grazing-incidence small-angle X-ray scattering (GISAXS) study of the PLD growth of Pt interlayer on sapphire (0001) will be published elsewhere [20].

After the growth completion, the XRD and XRR measurements were also performed before and after cooling the sample down to RT. The XRR and XRD reciprocal-space maps of the scattered intensity were measured using a linear detector and acquiring individual detector frames for a sequence of incidence angles ω of the primary X-ray beam. From the XRR maps, specular X-ray reflectivities were extracted and the XRD maps have been measured around symmetric (222) and asymmetric (224) reciprocal-lattice points (RELPs) of Pt as well as around symmetric (002), (004), and asymmetric (108) LFO RELPs.

### 2.2. Electron Microscopy

Electron-microscopic techniques, particularly SEM and transmission electron microscopy (TEM), were applied ex situ to the LFO/sapphire and LFO/Pt/sapphire samples to study both the topography of the deposited LFO layers and their structural and microchemical peculiarities in a cross section. As-prepared samples could directly be used for SEM inspection of the layer surfaces, which was performed in a combined focused-ion-beam (FIB)/SEM system of the type Helios G4 FX (ThermoFisher Scientific) at 5 kV accelerating voltage, using pieces of some mm${}^{2}$ in size of the deposited Al${}_{2}$O${}_{3}$ wafers. For imaging of the surface topography, the secondary-electron (SE) signal was used.

Cross section TEM lamellas were taken out of the LFO/sapphire samples by ion milling that was also utilized by means of the Helios G4 FX. The typical dimensions of the lamellas were 15 μm width × 5 μm height × 50 nm thickness in transmission direction; of course, the TEM data were obtained from much smaller regions compared to XRD results. Since, first, FIB preparation experiments showed a more or less strong damage of the crystal structure of LFO layers, before FIB milling, the sample surfaces were coated with an extra protection layer, consisting of either 20 nm thick platinum or 80 nm carbon, respectively, using the Leica EM ACE600 sputter coater (Leica Microsystems). As usual for FIB preparation of TEM lamellas, on top of this protection layer, an additional Pt layer was deposited inside the FIB instrument. Coarse FIB milling was performed at 30 kV accelerating voltage with Ga${}^{+}$ ions, whereas the lamella surfaces were finally polished via ca. 10,000 scans at each side at 1 kV and 7 pA ion current. The lamellas were investigated by combined STEM/EDXS and HRTEM methods.

TEM studies were carried out by using a 200 kV FEI Tecnai Osiris and a 300 kV FEI Titan 80–300 transmission electron microscopes (ThermoFisher Scientific), which both are equipped with thermally assisted field-emission electron guns. Images were recorded by means of 4 mega-pixel charge-coupled device camera (CCD) UltraScan 1000 P (Gatan) positioned on the optical axis, and the camera was controlled by the DigitalMicrograph (Gatan) software. For each TEM image, the exposure time was in the order of 0.5 to few seconds. In more detail, the crystal structure of the LFO layers as well as its orientation relationship with respect to the adjacent substrate or Pt interlayer were studied by HRTEM in the Titan microscope. This microscope is equipped with a correction-lens system (in short, Cs image corrector) for spherical and other aberrations of the objective lens allowing to obtain HRTEM images with an information limit of 0.08 nm. The Cs image corrector was used to minimize and determine the residual objective-lens aberrations as described by Uhlemann and Haider [21]. In the Osiris microscope, STEM images were additionally taken by means of the high-angle annular dark-field (HAADF) detector, which yields atomic-number contrast (Z-contrast) images. The Osiris also has a so-called FEI ChemiSTEM detector with four silicon-drift-detectors (SDD) for high-efficiency EDXS analyses. With this EDXS system, two-dimensional element-specific maps were recorded in the STEM mode. For X-ray mapping, the measuring time amounted to typically 1–1.5 h and any possible specimen drift was corrected by the Esprit software (Bruker). This software was also used for quantification of the obtained EDXS data by means of the thin-film approximation [22].

## 3. Results

In this section, we present and discuss in situ XRD and XRR as well as ex situ electron microscopy results.

### 3.1. Analysis of X-ray Scattering Data

**Figure 1:**
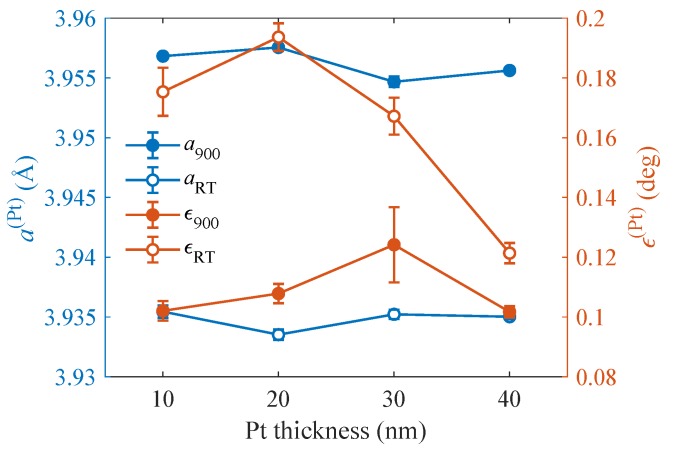
The Pt lattice parameters $a^{\left(\mathrm{Pt}\right)}$ and $\epsilon^{\left(\mathrm{Pt}\right)}$ determined from 2D reciprocal-space mapping (RSM) around the Pt reciprocal-lattice points (RELPs) 222 and 224 at room temperature (RT) and at the growth temperature of 900$\;{}^{\circ }\;C$.

Prior to LFO growth, the lattice parameters of the Pt layer were determined by measuring the RSMs around the symmetric 222 and asymmetric 224 RELPs of Pt both at RT and at the growth temperature $T_{d}=900\;{}^{\circ }\;C$. Since the Pt surface is (111) and the Pt layer is biaxially strained, the Pt lattice is assumed rhombohedral with the parameters $a^{\left(\mathrm{Pt}\right)}$ and rhombohedral distortion angle $a^{\left(\mathrm{Pt}\right)}$ ($\epsilon^{(\mathrm{Pt})=0}$ means cubic lattice). The results are shown in Figure 1. From the figure, it is obvious that the lattice parameter *a* is almost independent from the Pt thickness and exhibits an expected temperature dependence due to thermal expansion. On the other hand, the RT values of the rhombohedral distortion strongly depend on the Pt thickness.

**Figure 2:**
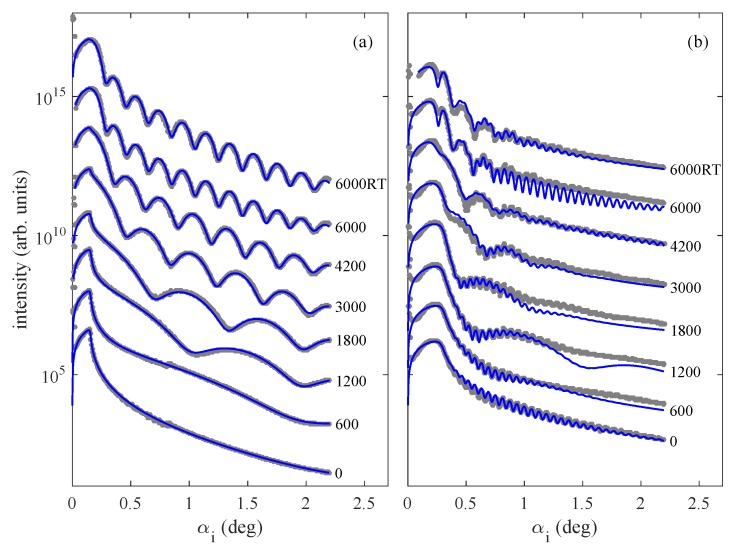
X-ray reflectivity curves taken during pulsed-laser deposition (PLD) growth: the parameters of the curves are the total numbers of laser shots of the LFO deposition. The measurements have been performed at the growth temperature of 900$\;{}^{\circ }\;C$, and the curves denoted “RT” have been acquired after sample cooling to room temperature. We show the X-ray specular-reflection (XRR) curves of the *h*-LuFeO${}_{3}$ (LFO) layers deposited directly on sapphire (**a**) and on a 40 nm thick Pt layer (**b**). The dots and full lines represent the measured and fitted curves, respectively; the curves are shifted vertically for clarity.

The XRR curves measured after individual LFO growth steps were fitted to a standard model of a multilayer with rough interfaces [23]. From the fit, we determined the thicknesses of the Pt and LFO layers, their densities, and root-mean square (rms) roughnesses of the interfaces. As an example, in Figure 2, we present the XRR curves taken during the LFO deposition directly on the sapphire substrate (Figure 2a) and on a Pt layer with the nominal thickness of 40 nm (b). The XRR curves of Pt0 and Pt40 display different behaviors. The fitting of the XRR curve before the LFO growth, marked “0” in Figure 2b, enables us to determine the thickness of the Pt interlayer grown for Pt40. A similar procedure was applied for the other samples Pt10, Pt20, and Pt30.

**Figure 3:**
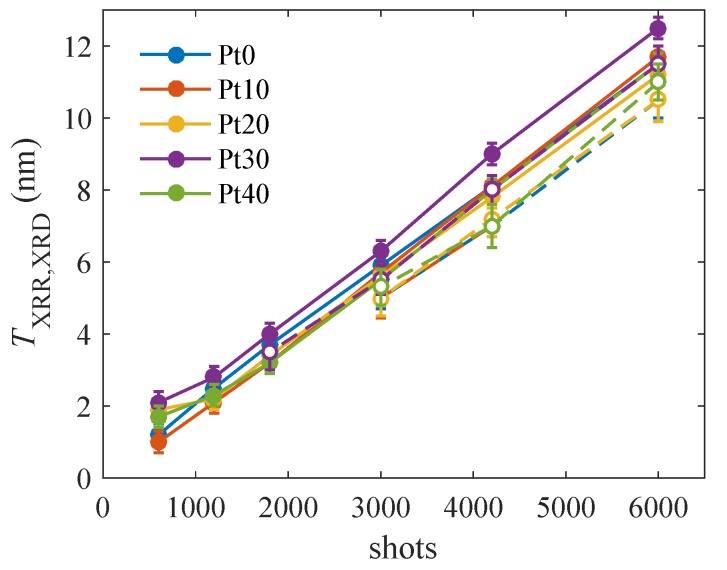
The LFO thicknesses $T_{\mathrm{XRR}}$ (full symbols) and $T_{XRD}$ (empty symbols) determined from XRR and XRD data as functions of the number of laser shots: The parameter of the curves is the nominal thickness of the Pt layer underneath in nm.

Figure 3 displays the evolution of the LFO thicknesses $T_{\mathrm{XRR}}$ and $T_{\mathrm{XRD}}$ determined from XRR and XRD measurements during the deposition. From the figure, it is obvious that the LFO thickness grows indeed linearly with the number of shots and that the growth rate is almost not dependent on the thickness of the underlying Pt layer. The estimated growth rate for LFO was found in the range of 0.555 nm/min to 0.62 nm/min for the different samples. One can conclude that the growth rate is almost not dependent on the thickness of the underlying Pt layer. Unfortunately, the rms roughnesses determined from the XRR data are burdened with a large statistical error so that their values are not conclusive.

**Figure 4:**
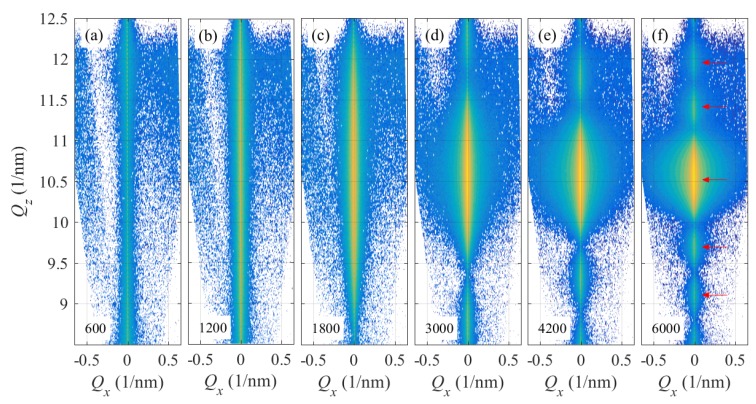
The XRD reciprocal-space maps taken around the LFO reciprocal-lattice point (002); the panels (**a**–**e**) display the reciprocal-space maps taken after individual growth steps; the parameters of the panels are the numbers of the laser shots. The maps were measured in situ during the LFO growth on a 40 nm Pt interlayer (sample Pt40). The intensities are displayed logarithmically, and the colors span over 4 decades. The red arrows in panel (**f**) indicate the side maxima along the $Q_{z}$ discussed in the text.

**Figure 5:**
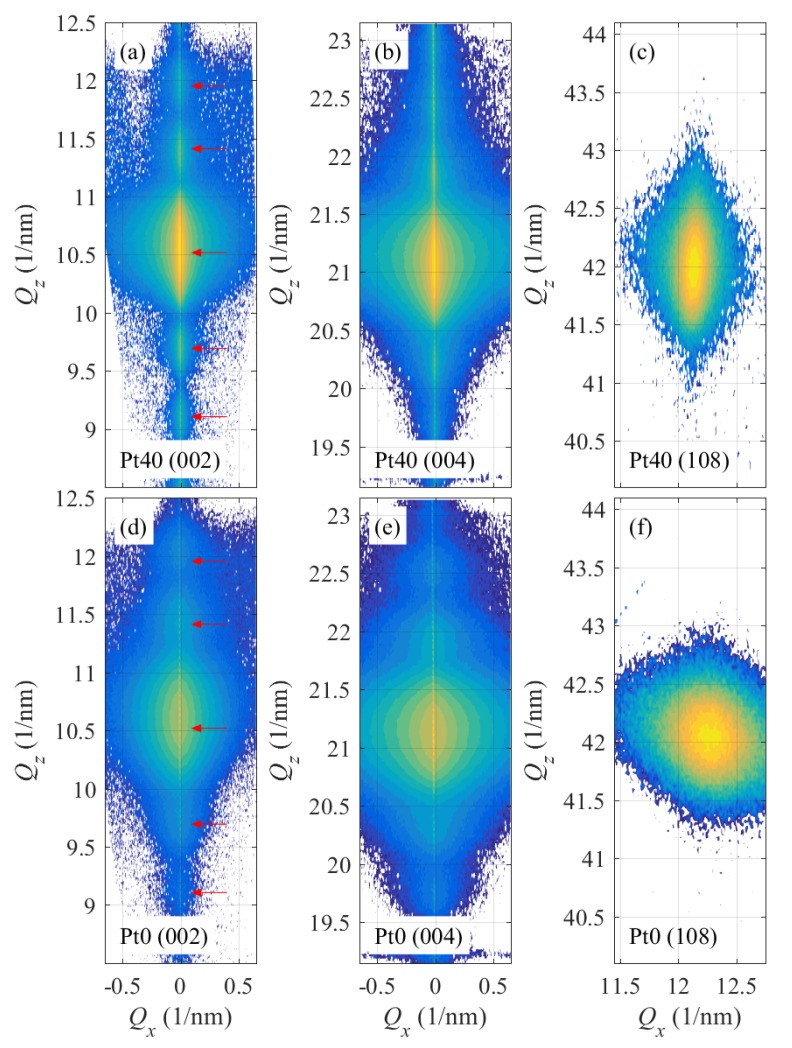
The XRD reciprocal-space maps taken around the LFO reciprocal-lattice points (002) in panels (**a**,**d**), (004) in (**b**,**e**), and (108) in panels (**c**,**f**). The maps were measured after the completion of the LFO growth on a naked sapphire substrate [sample Pt0, (**d**–**f**)] and on 40 nm Pt interlayer [sample Pt40, (**a**–**c**)]. The intensities are displayed logarithmically, and the colors span over 4 decades. The red arrows in panels (**a**,**d**) indicate the side maxima along the $Q_{z}$ discussed in the text.

Figure 4 and Figure 5 present typical examples of the XRD measurements. Figure 4 shows the evolution of the RSMs taken around the (002) RELP taken in situ during the LFO deposition on a 40 nm thick Pt interlayer (sample Pt40). With increasing number of laser shots (parameters of the figures), the maximum diffracted intensity increases, its width along $Q_{z}$ obviously decreases, and the $Q_{x}$-width slightly increases. This behavior will be studied in detail later, using horizontal and vertical cross sections of the diffraction maxima.

The XRD reciprocal-space maps around various LFO reciprocal-lattice points measured after the LFO growth (6000 laser shots) of samples Pt0 and Pt40 are displayed in Figure 5. The comparison of XRD-RSMs of Pt0 and Pt40 drawn in Figure 5 for the same range of scattering vectors Q reveals different important features which qualitatively shows the effect of Pt interlayer on the structure of LFO. The presence of well-defined oscillations in the $Q_{z}$ cut (side maxima) in the (002) RSM reflection denoted by red arrows in Figure 5a indicates a sharp (abrupt) interface in the case of Pt40 between the LFO and Pt layers. The same effect can be seen also in the case of 6000 shots for Pt40 on Figure 4e. The distance separating the side maxima enables us to determine the film thickness of LFO layer. Oppositely, the (002) RSM in Figure 5d shows less visible side maxima in Pt0 (a smearing effect). This is probably connected to a smeared interface between the LFO layer and the Al${}_{2}$O${}_{3}$ substrate and to the disturbance of the crystalline structure. This effect will be better visible in the $q_{z}$ cuts discussed later in detail using the simulation of the RSMs of (004) and (108) reflections.

The analysis of the XRD RSMs is based on the mosaic-block model published previously in Reference [23,24]. Within this model, the diffracting layer consists of randomly rotated and randomly placed mosaic blocks and we assume that the measured intensity is averaged over a statistical ensemble of all block configurations (ergodicity). The ergodicity assumption is justified, since the irradiated sample volume is much larger than the coherently irradiated volume (approx. 1 μm${}^{3}$), and the mosaic blocks are usually much smaller than 1 μm.

The diffracted intensity is expressed as a sum of coherent and diffuse parts:

(1) $$I_{h}\left(q\right)=I_{h}^{\mathrm{coh}}\left(q\right)+I_{h}^{\mathrm{diff}}\left(q\right)={\left|\langle E_{h}\left(q\right)\rangle \right|}^{2}+\langle {\left|E_{h}\left(q\right)-\langle E_{h}\left(q\right)\rangle \right|}^{2}\rangle .$$

Here, we denoted that $q=Q-h,\;Q=K_{f}-K_{i}$, $K_{i,f}$ are the wave vectors of the incident and scattered radiation, that Q is the scattering vector, that $E_{h}\left(q\right)$ is the amplitude of the diffracted radiation in reciprocal point q, and that h=(002),(004),(008) or (108) is the reciprocal-lattice vector. The averaging 〈〉 is performed over all sizes and orientations of the blocks.

Performing the averaging, we find that $I_{h}^{\mathrm{coh}}\left(q\right)=0$ is zero, i.e., the scattered radiation contains only the diffuse component. In the calculation of the diffuse component, we assume that the mosaic blocks are cylindrical with the height *H*, H≤T, where *T* is the layer thickness. The block diameters are random with the mean value *D* and rms deviation $\sigma_{D}$. The crystal lattices of the blocks are randomly rotated from their nominal orientation, and the random rotations are normally distributed with the rms dispersion Δ.

In Reference [24], we have derived the following expression for the diffusely scattered intensity (neglecting absorption in the layer)

(2) $$I_{h}^{\mathrm{diff}}\left(q\right)=A\int_{V}d^{3}rP\left(r\right)\exp\left(-\frac{\Delta^{2}}{6}{\left|h\times r\right|}^{2}\right)e^{-iq.r}.$$

Here, we denoted r≡(x,y,z) as a point in the LFO layer; the integration is performed over the volume *V* of the layer, z∈[−T,0]. The *z*-axis is perpendicular to the sample surface, and *A* is a prefactor containing the structure factor of the LFO lattice, irradiated sample volume, and the primary intensity, among others. The function P(r) is the probability that two points (0,0,0) and r lie in the same block. An explicit expression for this probability is quite cumbersome; it contains *H*, *D*, and $\sigma_{D}$ as parameters.

The lateral width of the diffraction maximum is affected both by *D* and Δ. If Δ=0, the width is inversely proportional to the mean block diameter *D*; this broadening is independent from the reciprocal-lattice vector h. On the other hand, the broadening due to the misorientation Δ is always perpendicular to h and the width is proportional to |h|. This fact makes it possible to distinguish both broadening effects by comparing the diffraction maxima measured around various h’s. The finite layer thickness *T* gives rise to additional maxima along $q_{z}$; their distance is inversely proportional to *T*. In addition, the vertical width of the main maximum is also inversely proportional to the mean block height *H*.

**Figure 6:**
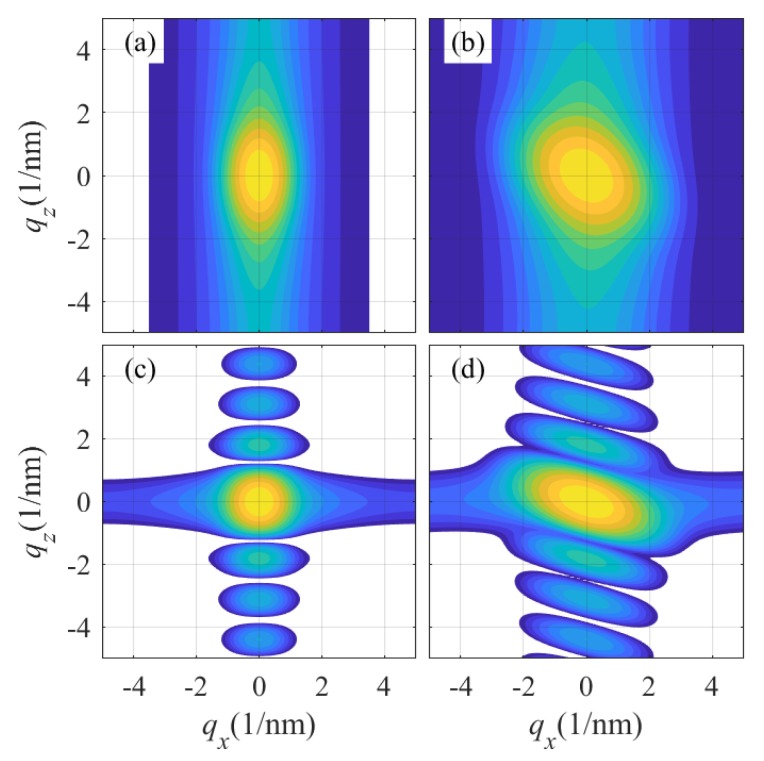
Reciprocal-space maps of a mosaic LFO layer simulated in symmetrical (004) (**a**,**c**) and asymmetrical reflection (108) (**b**,**d**). In panels (**a**,**b**), the heights *H* of the mosaic blocks are random and smaller than the layer thickness *T*; in (**c**,**d**), the blocks penetrate the whole layer thickness. See the main text for the simulation parameters. The intensities are plotted logarithmically; the colors cover 4 decades.

Figure 6 presents examples of reciprocal-space maps of a mosaic layer simulated for symmetrical (004) (Figure 6a,c) and asymmetric (108) reflections (Figure 6b,d), assuming the same mean lateral block diameters D=20 nm and the same rms misorientation Δ=2 deg. The diameters of the blocks are randomly distributed assuming Gamma distribution with the rms dispersion $\sigma_{D}=4$ nm. The layer thickness was set to T=5 nm; in Figure 6a,b, the mean block height was H=4 nm (i.e., smaller than the layer thickness) with the rms dispersion $\sigma_{H}=1$ nm, and in Figure 6c,d, H=T was assumed. From the graphs, it is obvious that the shape of the diffraction maximum is affected indeed by the diffraction asymmetry. If all mosaic blocks penetrate through the whole layer thickness (H=T), the reciprocal space maps exhibit side maxima along the vertical $q_{z}$-axis; from their distance, the layer thickness can be determined. The side maxima disappear if H<T; in this case, the mean block height *H* can be estimated from the vertical maximum width.

**Figure 7:**
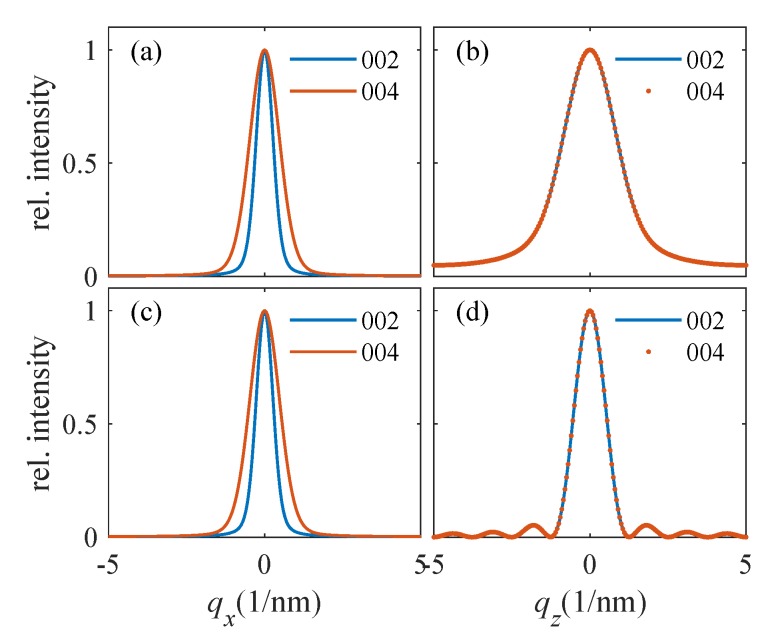
Horizontal $q_{x}$-cuts (**a**,**c**) and vertical $q_{z}$-cuts (**b**,**d**) of diffraction maxima of a mosaic LFO layr calculated in symmetrical diffractions 002 and 004: Other simulation parameters are the same as in Figure 6. Panels (**a**,**b**) and (**c**,**d**) display the maxima for H<T and H=T, respectively.

Figure 7 compares the horizontal (Figure 7a,c) and vertical cuts (Figure 7b,d) of the reciprocal-space maps calculated for the same parameters as in Figure 6 and two symmetrical reflections h=(002),(004). The widths of the horizontal cuts is proportional to the length of the diffraction vector h, and the shape of these cuts depends on the statistical distribution of the block misorientations. If the misorientations are normally distributed as assumed in Equation (2), the profile of the cut is Gaussian; however, actual distribution of the block misorientations can be quite different. From the figure, it also follows that the lateral cuts are not dependent on the vertical block size, that the vertical cuts of the diffraction maxima are identical for all symmetrial reflections, and that they do not depend on the block misorientation. This is due to the assumption that the crystal lattices of individual blocks are only randomly rotated and not randomly strained, i.e., the lengths of the reciprocal lattice vectors in different blocks are the same.

**Figure 8:**
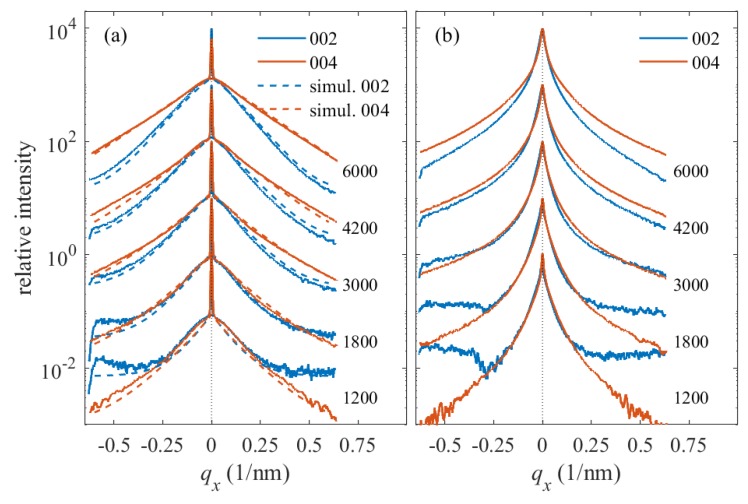
Experimental (full) and simulated (dashed lines) $q_{x}$ cuts measured in symmetrical diffractions 002 and 004 during the LFO deposition: the parameters of the curves are the numbers of the laser shots. Panels (**a**,**b**) display the $q_{x}$ cuts measured on samples PT0 and Pt40, respectively. The curves are shifted vertically for clarity.

In Figure 8, we plot the examples of $q_{x}$-cuts of the 002 and 004 symmetrical diffraction maxima measured during the LFO deposition of samples Pt0 (Figure 8a) and Pt40 (Figure 8b). The diffraction maxima in Figure 8a exhibit a very narrow central peak and a broad component; the width of the central peak is comparable to the angular resolution of the experimental setup. This peak corresponds to the narrow vertical streaks in Figure 5, and it is a tail of a very narrow substrate maximum; this maximum is vertically elongated (crystal truncation rod—CTR) and extends from the sapphire RELP to the LFO diffraction maximum. The presence of a distinct narrow CTR makes it possible to separate the substrate contribution from the LFO maximum and to fit the measured horizontal cuts to the mosaic model explained above. From the figure, it is also obvious that the shape of the horizontal cuts differs from the theoretical shapes in Figure 7; therefore, the distribution of the block misorientations is far from Gaussian. In order to include this fact, we modified Equation (2) to the following *ad hoc* form

(3) $$I_{h}^{\mathrm{diff}}\left(q\right)=A\int_{V}d^{3}rP\left(r\right)\exp\left[-\frac{1}{6}{\left(\Delta \left|h\times r\right|\right)}^{p}\right]e^{-iq.r},$$

and parameter *p* was fitted. Then, strictly speaking, Δ is no more the rms misorientation. However, its value can still be used for the assessment of the degree of mosaicity of the LFO layer. We have fitted the 002 and 004 $q_{x}$-cuts together, which improved the robustness of the fit, and we excluded the central narrow CTR peak from the fit; the fitted curves are plotted by dashed lines. From the fit, we determined Δ and *D*, and the simulated curves were almost insensitive to the rms deviation $\sigma_{D}$; we have set this parameter to $\sigma_{D}=0.3D$.

Unfortunately, the $q_{x}$-cuts in Figure 8b do not exhibit distinct narrow CTR spikes and both the CTR and the broad diffuse maximum are merged. This fact makes a direct fitting similar to Figure 8a impossible. Nevertheless, we determined the full widths of the cuts at the level of 5% of the maximum intensity; from Figure 8a, it follows that this intensity level roughly corresponds to the half maximum of the diffuse intensity contribution. Comparing these widths with simulations, we were still able to estimate the Δ and *D* values. From the fitting of the measured lateral cuts along $q_{x}$ with the simulated diffracted curves given by from Equation (3) (see Figure 8), the mean sizes of mosaic blocks were derived for the different growth steps (i.e., as function number of shots). It should be emphasized that the lateral broadening is assumed to be mainly due to lateral size of the blocks and not to any kind of defects.

**Figure 9:**
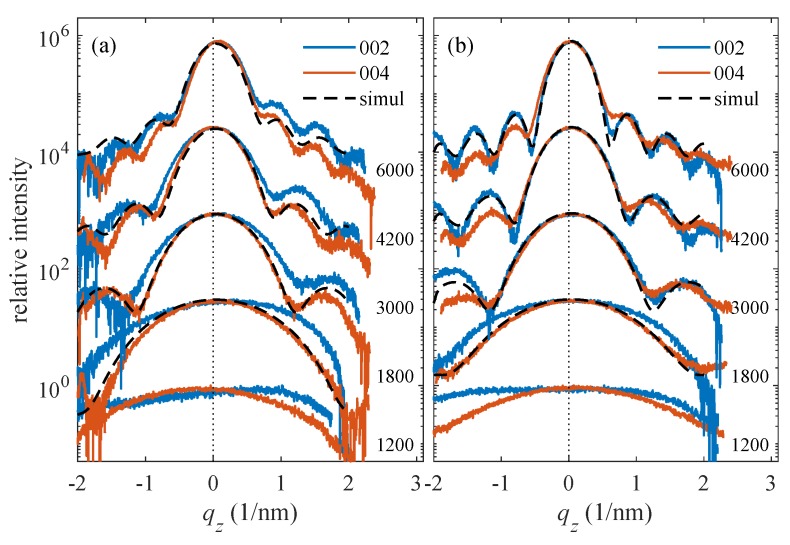
The same situation as in Figure 8, vertical $q_{z}$-cuts. Panels (**a**,**b**) show the data measured on samples Pt0 and Pt40, respectively.

The vertical $q_{z}$-cuts of the (002) and (004) diffraction maxima of samples Pt0 and Pt40 are plotted in Figure 9a,b, respectively, along with the fits. The experimental cuts were extracted from the measured reciprocal-space maps along a vertical line at $q_{x}=0.02$ nm${}^{-1}$, i.e., just aside from the CTR streak. The measured cuts exhibit distinct side maxima, from which the corresponding thicknesses $T_{\mathrm{XRD}}$ were determined; their values are plotted in Figure 3. Since $T_{\mathrm{XRD}}$s are only slightly smaller than the thicknesses $T_{\mathrm{XRR}}$, we conclude that the mosaic block heights are comparable to the whole LFO thickness. Further, especially for thicker LFO layers, the widths of the $q_{z}$-cuts for (002) and (004) maxima are the same; this fact indicates that the LFO lattices in individual mosaic blocks are indeed not strained. The widths differ for smaller LFO thicknesses in all samples Pt0–Pt40; in this case, the 002 widths are *larger* than the 004 ones, which excludes random strains in the LFO lattices, too. The reason of this difference is not clear; most likely, the sample volume irradiated by the primary X-ray beam is larger for 002 than in 004 so that the former diffraction maximum could be more sensitive to larger sample inhomogeneities.

From the $Q_{z}$-positions of the 002, 004, and 008 maxima, we determined the out-of-plane lattice parameter *c* of LFO; for the correction of the misalignment of the vertical sample position, we used the well-known Cohen–Wagner approach, in which the out-of-plane lattice parameters *c* determined from individual diffraction maxima were plotted vs. cotan(Θ)cos(Θ) (2Θ is the scattering angle) and the resulting linear dependence was extrapolated to zero [25]. From the slope of this linear dependence, we determined the value of the sample misalignment, which we take into account in the determination of the in-plane lattice parameter *a* of LFO from the asymmetric diffraction maximum 108.

**Figure 10:**
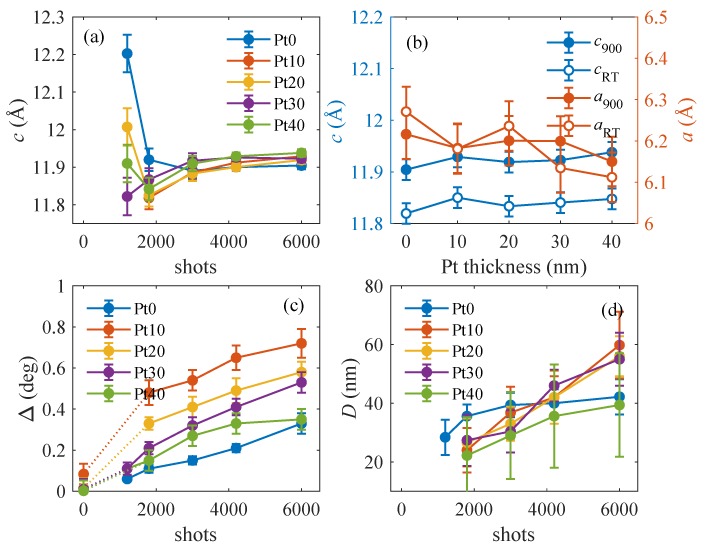
Results of the fitting of the $q_{x}$-cuts: (**a**,**c**,**d**) the dependences of the vertical lattice parameter *c*, rms misorientation Δ, and mean block diameter *D* on the LFO thickness expressed by the numbers of laser shots on the horizontal axis. The points plotted for 0 shots present the values of Δ of the Pt layer, the parameters of the curves are the Pt thicknesses underneath. Panel (**b**) shows the LFO lattice parameters *a* and *c* measured after the growth completion as functions of the thickness of Pt interlayer. The room-temperature (RT) and 900$\;{}^{\circ }\;C$ data are displayed as empty and full symbols, respectively.

The results of the fits are summarized in Figure 10. In Figure 10a, we plotted the variation of the out-of-plane LFO lattice parameter with the LFO thickness for the samples Pt0, Pt10, Pt20, Pt30, and Pt40 as measured after the aforementioned growth steps at the deposition temperature $T_{d}=900\;{}^{\circ }\;C$. The values are determined with a quite large error; however, the overall tendency is obvious—above approx. 3000 laser shots the LFO lattice parameter *c* remains almost constant. From this fact, we conclude that, at the beginning of the LFO deposition, the LFO layer is elastically strained and that this strain gradually releases during first 3000 shots, i.e., at the LFO thicknesses up to approx. 5 nm.

However, the final *c* parameter of about 11.92 Å is still much larger than the nominal value of the strain-free lattice parameters (a=6.0057 Å, c=11.6767 Å) of *h*-LuFeO${}_{3}$ (as obtained by density functional theory calculations under the condition of zero pressure). Similar results were found by Jeong et al. [9], who demonstrated that hexagonally constrained LuFeO${}_{3}$ epitaxial films are compressively strained along the in-plane direction while it is under a tensile strain along the out-of-plane (thickness) direction. The *c* parameters of thinner LFO layers are burdened by large errors since the diffraction maxima are quite broad along $Q_{z}$. These widths are larger than those following from the layer thickness, i.e., the LFO layers exhibit inhomogeneous strain in the vertical direction.

Figure 10b displays the dependence of the LFO in-plane (*a*) and out-of-plane (*c*) lattice parameters on the nominal Pt thickness, measured after the growth completion at the growth temperature of 900$\;{}^{\circ }\;C$ and at RT. The accurate determination of the in-plane lattice parameter *a* was performed from the RSMs of the (108) reflection when the number of shots exceeds 3000 shots (i.e., about 5 nm) due to the weakness of diffracted intensities of LFO (108). As stated in the previous paragraph, the out-of-plane *c* values are almost independent from the Pt thickness. The measurement errors of the *a* values are large; however, a general tendency is obvious—the *a* parameter slightly decreases with increasing Pt thickness from a=6.2161 Å for Pt0 to a=6.15 Å for Pt40 and aims to the strain-free in-plane lattice parameter of a=6.0057 Å. The values of the out-of-plane parameters *c* are very comparable to the ones determined by Disseler et al. [3] (a=6.050 Å, c=11.97 Å) measured by XRD in the case of a MBE-grown *h*-LFO film on Al${}_{2}$O${}_{3}$ with a thickness of 70 nm without interlayer. However, our in-plane lattice parameters are slightly larger, indicating that our *h*-LFO films are more relaxed in the surface-plane direction.

Figure 10c shows the variation of the rms misorientation Δ with the LFO and Pt thicknesses (i.e., numbers of shots); the points plotted for zero shots represent the values of Δ of the Pt layers prior to LFO growth. Interestingly, the Δ *increases* with increasing LFO thickness, which can also be explained by increasing degree of elastic relaxation and consequently increasing degree of buckling of the LFO (001) basal planes. The dependence of Δ on the Pt thickness is related with the morphology of the Pt layer investigated by SEM, which changes from an island structure in Pt10 to a more uniform morphology for Pt40. This means that the island-like morphology enhances the misorientation of the LFO mosaic blocks, as it will be demonstrated by STEM/EDXS and HRTEM. Furthermore, the smallest Δ’s were achieved in the layers deposited directly on sapphire Pt0, but with increasing Pt thickness, Δ decreases and it converges to the value found in the case of Pt0, where the surface morphology is smooth.

Finally, in Figure 10d, we plotted the mean lateral block diameters *D*. The data are weighed down by errors; however the general tendency is visible—*D* increases with increasing LFO thickness in similar way as in the case of the rms misorientation Δ. In addition, *D* in the case of Pt40 approaches the value of Pt0. This behavior could be explained by the change in the surface morphology of the film, which becomes smooth due to the collapse of Pt islands as the Pt thickness increases to 40 nm.

In order to assess the degree of lattice relaxation of LFO, we compare the LFO lattice parameters with the lattice parameter of the Pt interlayer. Since, as we show in the next section, the direction $\left[2\bar{1}\bar{1}0\right]$ in *h*-LFO is parallel to $\left[1\bar{1}0\right]$ Pt, we compare the Lu–Lu (or Fe–Fe) distance along $\left[2\bar{1}\bar{1}0\right]$ in *h*-LFO, being $a^{\left(\mathrm{LFO}\right)}$ with the double Pt–Pt distance $d^{\left(\mathrm{Pt}\right)}$ along $\left[1\bar{1}0\right]$ Pt. The LFO/Pt lattice misfit is then $[a^{\left(\mathrm{LFO}\right)}-d^{\left(\mathrm{Pt}\right)}]/d^{\left(\mathrm{Pt}\right)}$. Assuming the abovementioned rhombohedral distortion of the Pt lattice, the double Pt–Pt distance is

$$d^{\left(\mathrm{Pt}\right)}=a^{\left(\mathrm{Pt}\right)}\left(1-\sin\epsilon^{\left(\mathrm{Pt}\right)}\right)\sqrt{2}.$$

**Figure 11:**
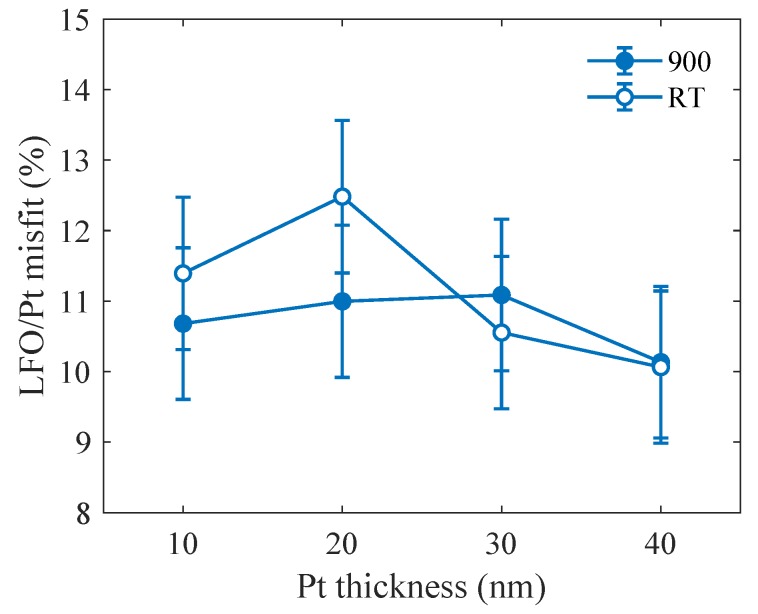
The LFO/Pt lattice misfit as function of the Pt thickness measured at the growth temperature of 900$\;{}^{\circ }\;C$ (full) and at room temperature (empty points).

Using the Pt lattice parameters shown in Figure 1 and the LFO lattice parameter $a^{\left(\mathrm{LFO}\right)}$ from Figure 10, we calculated the dependence of the lattice misfit on the Pt thickness; the results are in Figure 11.

The mean value of the LFO/Pt lattice misfit at $T_{d}=900\;{}^{\circ }\;C$ is about 10% while it is about 29% in the case of LFO/Al${}_{2}$O${}_{3}$. There is no remarkable variation of the lattice misfit with the Pt thickness, but there is a significant reduction of the lattice misfit between LFO and Al${}_{2}$O${}_{3}$ by introducing the Pt interlayer. The influence of the misfit will be discussed in the next chapter.

### 3.2. Characterization of the LFO/Pt Layers by Electron-Microscopic Techniques

To get information about the topographical peculiarities of the LFO layers as well as about their thicknesses and crystal structure in real space, the LFO/sapphire and LFO/Pt/sapphire samples were also characterized by SEM and TEM techniques. In this section, we present only the typical examples of samples Pt0, Pt20, and Pt40.

**Figure 12:**
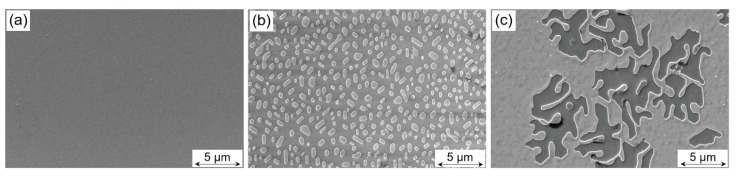
Secondary electron images of the sample surfaces as revealed by their SEM inspection at 30 kV: (**a**) LFO layer deposited directly on sapphire (Pt0), (**b**) on a nominally 20 nm thick Pt layer (PT20), and (**c**) on a Pt layer of 40 nm nominal thickness (Pt40).

**Figure 13:**
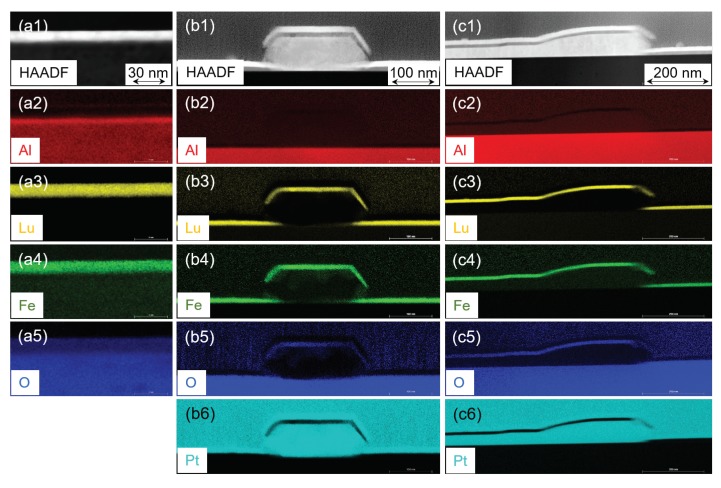
Combined STEM/energy-dispersive X-ray spectroscopy (EDXS) analysis of the element distribution in cross sections of the LFO/Pt layers on sapphire: STEM high-angle annular dark-field (HAADF) image and X-ray maps of the distribution of the elements Al, Lu, Fe, O, and Pt for the LFO layer directly deposited on sapphire (sample Pt0) (panels **a1**–**a5**), on top of the 20 nm Pt layer (sample Pt20) (**b1**–**b6**), and on the 40 nm Pt layer (sample Pt40) (**c1**–**c6**). The individual energy-dispersive X-ray data were quantified by using the thin-film approximation, and the noise of the obtained maps was reduced by applying a 3 × 3 mean filter.

Figure 12 shows the results of the SEM inspection of samples Pt0, Pt20, and Pt40. Here, SE images are depicted of the sample surfaces, i.e., the LFO layers are visualized in top view. By comparing the visible contrast features obtained from the sample surfaces, clear differences can be seen for the individual LFO layers. In more detail, sample Pt0 in Figure 12a exhibits an extremely smooth surface. Unlike this, samples Pt20 and Pt40 reveal a quite different surface topography. In the case of sample Pt20, some underlying area is evidently covered with island-like objects and there is free space everywhere in between them. Typically, the islands have a minimum dimension of approximately 200 nm, whereas their longest amounts to about 1 μm. Moreover, there seems to be a crystallographic orientation alignment of elongated islands in a way that three main axes rotated by 120 deg exist. Probably, this orientation alignment is caused by the lattice misfit or tilt, respectively, of the two adjacent materials, namely one in the islands and the other in the underlying layer. Corresponding STEM/EDXS results of sample Pt20 in Figure 13(b1–b6) clearly prove that the islands are composed of platinum, and they are covered by an about 10 nm thick LFO layer. Therefore, the interaction of the crystal lattices of the face-centered cubic (fcc) Pt and the hexagonal Al${}_{2}$O${}_{3}$ substrate during growth is most likely the reason for the preferred orientation of the Pt islands. For sample Pt40, the surface coverage with platinum is much larger compared to sample Pt20. Here, the majority of the surface is covered by a continuous LFO layer, and only discrete holes in LFO and a few residual island-like LFO objects in these holes can be observed (cf. Figure 12c). For both samples Pt20 and Pt40, the height difference between the topmost level and the deepest regions seems to be several 10 nanometers. More precise values of the heights of Pt islands are found by TEM imaging, and they are given in the following paragraph.

To elucidate the setup of the different LFO/Pt layer stacks on sapphire from the view of their chemical composition, combined STEM/EDXS analyses were performed ex situ after layer growth. For this purpose, element-specific X-ray maps were recorded from FIB-prepared TEM lamellas. Typical results obtained for Pt0, Pt20, and Pt40 are depicted in Figure 13, which shows STEM HAADF images together with the corresponding color-coded X-ray maps of the element distribution of Al (red), Lu (yellow), Fe (green), O (blue), and Pt (turquois). These maps are the results of quantification of raw data using the thin-film approximation, i.e., the background contribution was subtracted and atomic-number (Z) correction was applied; however, absorption and fluorescence effects are not corrected.

The simplest configuration is shown in Figure 13(a1–a5), which is the sample constituted by the pure LFO layer deposited on top of sapphire (i.e., sample Pt0). The position of the LFO layer can clearly be seen in the corresponding Lu and Fe maps. Moreover, the LFO layer appears to be homogeneous over wide lateral dimensions and its thickness amounts to approximately 10 nm. Here, it should be noted that this LFO thickness of about 10 nm was determined for all three LFO/Pt samples, i.e., samples Pt0, Pt20, and Pt40. In contrast, for sample Pt20, the STEM/EDXS experiments revealed that no continuous Pt layer had formed during pulsed-laser deposition but rather an island-like growth can be observed. It must be noted that the topmost Pt layer visible in the STEM HAADF image [see Figure 13(b1)] by its high signal intensity as well as in the corresponding Pt map [Figure 13(b6)] was deposited by sputtering in order to protect the LFO layer during FIB milling; this is the same situation for sample Pt40. The height of the Pt islands is locally different; typical heights (excluding the LFO layer on top) are between approx. 70 nm and 100 nm. As one example, Figure 13(b1–b6) display a region with a Pt island of approximately 80 nm in height. Evidently, in cross sections, the Pt islands exhibit the shape of truncated pyramids. In regions without Pt islands, there is evidently no Pt interlayer in between LFO and Al${}_{2}$O${}_{3}$. In addition, because of the three-dimensional shape of the Pt islands, the LFO layer is interrupted on the side facets of the pyramid. This can particularly be concluded from the X-ray maps of the elements Lu and Fe [cf. Figure 13(b3,b4)]. In the case of sample Pt40 [Figure 13(c1–c6)], similar findings of an incomplete Pt interlayer in between sapphire substrate and LFO layer were made. For this LFO/Pt sample, because of its larger thickness of platinum compared to sample Pt20, higher heights of Pt islands in the range from approximately 145 nm to 220 nm could be measured. Moreover, in some regions, a Pt interlayer (not in the form of islands) was found in between the Al${}_{2}$O${}_{3}$ substrate and the PLD-grown LFO layer. Such a region, where obviously a layer-on-layer growth of LFO on Pt occurred, is depicted in the main field of view of Figure 13(c1–c6). Besides, in the right quarter of the imaged region, there is a zone with a missing Pt layer present.

The findings of STEM/EDXS analyses help understand the topographical features visible in the SE images of the different sample surfaces. With other words, for sample Pt0, the corresponding surface must show up nearly unstructured since it consists of a pure LFO layer. Contrary to this, in the case of sample Pt20, there are individual well-separated Pt islands which, together with the regions in between of them, are covered with LFO. As to sample Pt40, this sample is comprised of two different regions, namely one where a layer-on-layer growth of platinum and LFO on the sapphire occurred and another with a missing Pt interlayer but with Pt islands having the LFO layer on top. The latter region of island-like growth can be seen in the central area of the SE image in Figure 12c, whereas the adjacent outer areas show the LFO/Pt layer-on-layer growth and additionally the residual regions with dark contrast show the LFO directly grown on Al${}_{2}$O${}_{3}$.

**Figure 14:**
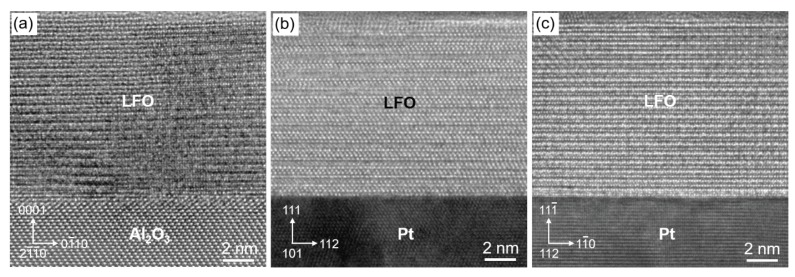
Structural characterization of the LFO layers and their orientation relationships with respect to adjacent sapphire substrate or Pt layer, respectively, by high-resolution transmission electron microscopy (HRTEM) imaging: LFO layer deposited directly on sapphire (**a**), on a nominally 20 nm thick Pt layer (**b**), and on a Pt layer of 40 nm nominal thickness (**c**).

Moreover, HRTEM imaging was done on the LFO layers to correlate the obtainable structural data with those of XRD measurements and to check for their epitaxial growth. For samples Pt0, Pt20, and Pt40, typical findings are summarized in Figure 14, in which only small transition regions between the LFO layer and the underlying material, i.e., sapphire or platinum, respectively, are shown. Generally, for each individual LFO/Pt sample, a hexagonal crystal structure was proved for lutetium iron oxide. However, there are clear differences in the crystalline quality of the LFO layers that mainly seems to depend on the adjacent material below. In detail, having a closer look at HRTEM images recorded from sample Pt0 (cf. Figure 14a), local image-contrast variations on the scale of few 10 nm attract attention; a more or less blurring of contrast features within the LFO layer can be seen. Probably, these phenomena are due to the high misfit in the order 29 % between the lattices of hexagonal (rhombohedral) *h*-Al${}_{2}$O${}_{3}$ and hexagonal *h*-LuFeO${}_{3}$, leading to a partly strongly disturbed crystal structure of the LFO layer. Despite these issues, the HRTEM image of Figure 14a gives insight into the structural arrangement of both crystal lattices, i.e., sapphire and LFO, on the atomic scale. As concluded from digital image analysis by means of fast Fourier transformation (FFT), the sapphire substrate (space group R-3cH: a=b=4.7607 Å, c=12.9947 Å; α=β=90 deg, γ=120 deg) may be orientated here with its $\left[2\bar{1}\bar{1}0\right]$ zone axis (ZA) parallel to the electron beam, and the *h*-LFO (space group P6${}_{3}$cm: a=b=5.96522 Å, c=11.70219 Å; α=β=90 deg, γ=120 deg) has the same ZA orientation; just as well, it could be the case that $\left[11\bar{2}0\right]$*h*-Al${}_{2}$O${}_{3}$‖$\left[11\bar{2}0\right]$ *h*-LFO. In each case, the out-of-plane orientation of the two crystal lattices is that the (0001) planes of *h*-Al${}_{2}$O${}_{3}$ are oriented parallel to the (0001) *h*-LFO planes. At the interface between sapphire and LFO layer, the latter must be compressed since the inter-planar distance $d_{\left(0\bar{3}30\right)}$ of LFO being 1.77 Å is larger than $d_{\left(0\bar{3}30\right)}$ of *h*-Al${}_{2}$O${}_{3}$ (1.37 Å). This explains that the out-of-plane lattice parameter *c* of approximately 12.0 Å is measured for the LFO lattice, which is larger than the strain-free value of 11.7 Å.

Already at first glance, the HRTEM images obtained from the LFO layers of samples Pt20 and Pt40 exhibit less disturbed crystal lattices. In addition, epitaxial relationships of the two crystal lattices, i.e., *h*-LFO and *fcc*-Pt, are more pronounced than for sample Pt0. In contrast to the latter, the image contrast observable of the LFO layer is relatively the same over a wide area. This noticeable difference is most likely due to the lower lattice misfit of about 6% between *h*-LFO and *fcc*-Ptt (space group Fm-3m: a=b=c=3.92316 Å; α=β=γ=90 deg). However, this value of the lattice misfit is affected by the elastic relaxation in thin sample lamellas and it cannot be directly compared with the misfit value determined from XRD RSMs on 2D samples.

In the case of Pt20, in the HRTEM image, the viewing direction is along the [101] zone axis of *fcc*-Pt that is parallel here to the $\left[10\bar{1}0\right]$ ZA of *h*-LFO. FFT analysis revealed that, in the out-of-plane direction, there is an epitaxial match of the (0001) lattice planes of LFO and the (111) Pt planes. In the in-plane direction, the $\left(1\bar{2}10\right)$ *h*-LFO planes are aligned parallel to the (112) Pt planes. For the present epitaxial arrangement between *h*-LFO and *fcc*-Pt, the LFO crystal lattice has the *c* lattice parameter of about (11.7±0.3) Å that is close to the bulk value. This also holds for sample Pt40 (see Figure 14c), the HRTEM image of which shows the Pt along its [112] ZA, whereas the LFO lattice is projected along the $\left[2\bar{1}\bar{1}0\right]$ ZA of *h*-LFO or its $\left[11\bar{2}0\right]$ ZA, respectively, being not distinguishable from each other. In a similar manner to Pt20, in the imaged field of view there is a well-developed orientation relationship between the crystal lattices of *h*-LFO and *fcc*-Pt, namely $\left(0\bar{1}10\right)$ *h*-LFO ‖$\left(1\bar{1}0\right)$ Pt [or $\left(1\bar{1}00\right)$ *h*-LFO ‖$\left(1\bar{1}0\right)$ Pt] for in-plane direction and $\left(11\bar{1}\right)$ Pt ‖ (0001) *h*-LFO for the out-of-plane case.

## 4. Discussion

The quality of the *h*-LFO crystalline layers was evaluated for the samples Pt0, Pt10, Pt20, and Pt40 by in situ XRD-RSMs during PLD growth at the growth temperature $Td=900\;{}^{\circ }\;C$ as well as after cooling to RT by XRD, STEM/EDXS, and HRTEM. The measured lateral cuts along $q_{x}$ given by Figure 8 shows that the broadening of the LFO diffracted maxima 002 and 004 as an indicative parameter for the crystal quality increases with number of shots. Moreover, the widths of the laterals cuts along $q_{x}$ measured after different growth decreases with increasing Pt thickness. This means that the presence of Pt interlayer plays an important role in improving the quality of LFO crystalline film. The disturbance of the crystalline structure, detected in the case of Pt0 at RT, was clearly demonstrated by HRTEM given in Figure 14. The vertical $q_{z}$-cuts of the 002 and 004 diffraction maxima of Pt40 have shown well-defined distinct side maxima in comparison with Pt0 (see Figure 9). The difference recorded in the behavior in the $q_{z}$-cuts of 002 and 004 could be the result of the disturbance in the LFO crystalline quality occurring during the growth of LFO directly on Al${}_{2}$O${}_{3}$ (i.e., Pt0).

SEM reveals different surface topographies for Pt0, Pt20, and Pt40. The combination STEM/EDXS has informed us about the growth mechanism of Pt layer, which led to island-like growth for Pt20 and a clear collapse of the island and increases in the degree of coverage for Pt40. The difference in the degree of coverage illustrated by SEM images in Figure 12b for Pt20 and Figure 12c for Pt40 does not affect significantly the out-of-planes and the in-planes lattice parameters as it could be demonstrated by XRD in Figure 10a,b. It should be emphasized that the LFO layer was grown on the Pt layer, having different morphologies for Pt10, Pt20, Pt30, and Pt40 as a more or less continuous layer. The presence of island-like morphology has affected the rms orientation of the LFO crystalline mosaic blocks. Furthermore, the collapse of the Pt islands with Pt thickness and the increase of the homogeneity of the Pt interlayer has drastically reduced the rms misorientation values Δ and the dimension of the LFO blocks, which approaches Pt0 values (see Figure 10c). This means that the rms LFO-block misorientation Δ was increased by the presence of Pt island.

The accurate determination of the in-plane lattice parameters from XRD-RSMs has enabled us to measure the lattice misfit, which was reduced from 29% in the absence of Pt interlayer to about 10% with the insertion of Pt interlayer. This has reduced the disturbance in the LFO crystalline structure. It should be noted here that the degree of coverage of Pt island has not alter conspicuously the lattice misfit values.

## 5. Summary

The structure quality of hexagonal LuFeO${}_{3}$ epitaxial layers grown by pulsed-laser deposition on sapphire without and with Pt interlayers of various thicknesses has been investigated by in situ high-resolution X-ray diffraction (reciprocal-space mapping) and by scanning electron microscopy (secondary electrons). Thin lamellas prepared by focused ion-beam milling were then inspected by high-resolution transmission electron microscopy as well as by scanning transmission electron microscopy together with energy-dispersive X-ray spectroscopy. The results clearly demonstrate that the structural quality of the LuFeO${}_{3}$ layers improves with the growing thickness of the Pt interlayer. This finding shows the possibility of deposition of multiferroic LuFeO${}_{3}$ on a bottom Pt electrode and opens the way for fabricating functional multiferroic structures.
